# EEG electrode digitization with commercial virtual reality hardware

**DOI:** 10.1371/journal.pone.0207516

**Published:** 2018-11-21

**Authors:** Christopher C. Cline, Christopher Coogan, Bin He

**Affiliations:** 1 Department of Biomedical Engineering, University of Minnesota, Minneapolis, MN, United States of America; 2 Department of Biomedical Engineering, Carnegie Mellon University, Pittsburgh, PA, United States of America; University of Tübingen, GERMANY

## Abstract

Accurate spatial co-registration of EEG electrode positions with individual head models is an important component for EEG source localization and imaging. Due to variations in head shape between individuals, this requires measurements of electrode locations in each individual. Existing hardware for digitization can be accurate, but also relatively expensive. With the goal of making digitization more accessible for a range of research laboratories, we have developed an open-source software tool that can make use of less expensive consumer virtual reality hardware for EEG electrode digitization. Here we describe our developed VRDigitizer system and compare it to existing digitization solutions. Experimental evaluations were performed in a phantom head model and in 12 human subjects. In our comparison experiments, VRDigitizer was able to measure electrode positions with a mean error of 3.74 mm, compared to 1.73 mm and 2.98 mm for the commercial systems tested.

## Introduction

Electroencephalography (EEG) uses electrodes on the scalp to noninvasively record electrical signals generated by the brain and conducted through the cerebrospinal fluid, skull, and scalp [1]. This process of volume conduction effectively blurs the EEG signals measured on the scalp, limiting the spatial resolution of these signals. Patterns of EEG activity observed on the scalp can be highly dependent on the cortical geometry of the underlying sources, and therefore analysis of EEG sensor activity is challenging with regard to its origins within the brain. In order to improve the spatial resolution of EEG, source imaging and localization approaches have been developed for estimating the underlying cortical source activity responsible for signals observed on the scalp, essentially projecting these signals back onto the brain [2–7]. A critical component of these approaches is the construction of a volume conduction model, which relates signals produced on the cortical surface to those measured by specific EEG electrodes on the scalp. Especially when using subject-specific head models for source imaging, it is critical to have an accurate estimate of where each EEG electrode was located on the subject’s head [8]. While standard EEG montages are widely used and allow approximate estimates of electrode locations, there can be large variability in head shape and cap placement across individuals and sessions. Therefore, it is preferable to actually measure the physical locations of the EEG electrode on individual subjects in order to build more accurate models for source imaging.

Various methods are available for digitizing, or measuring the 3D locations, of EEG electrodes on the head [9–16]. Hardware digitization solutions involve some method of 3D spatial measurements, such as pointing a tracked stylus at each electrode or photogrammetry-based methods that use images taken from multiple angles to reconstruct electrode location [12]. However, the cost of such digitization hardware has typically been on the order of $10,000, making these methods expensive for EEG research and broader applications.

In recent years, several consumer-oriented room-scale virtual reality (VR) systems have been introduced to the market. These systems, such as the HTC Vive, use 6 degree-of-freedom (DOF) tracking of a head-mounted display (HMD) and associated peripherals to provide immersive virtual reality experiences. Marketed for individual consumers, these systems currently cost less than $1000. Conveniently, the 6DOF tracking of VR controllers can be used for the same purpose as more specialized hardware digitization solutions for EEG electrode localization, at a significantly lower price.

**Fig 1:**
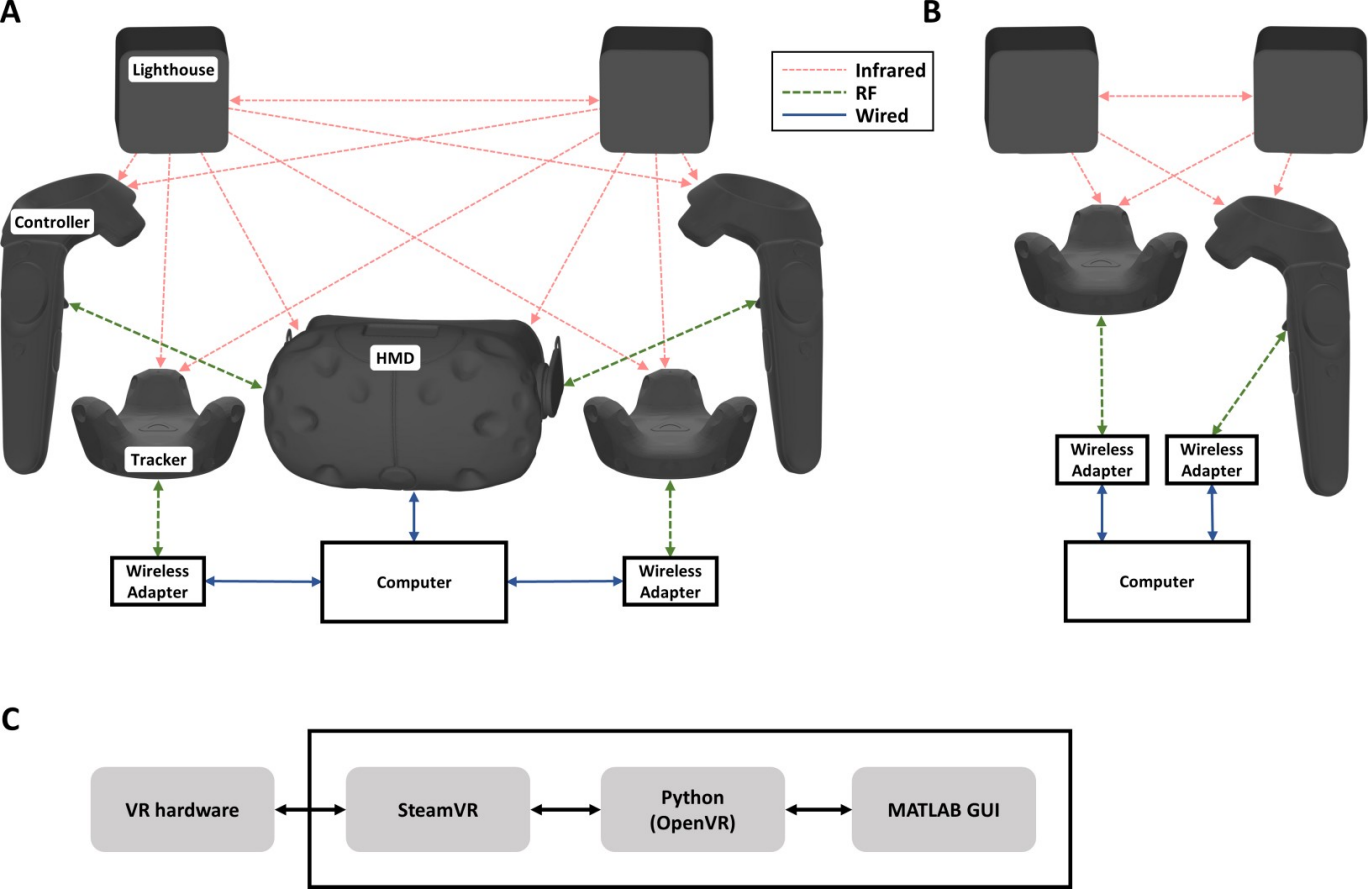
Diagrams of connections between hardware and software components of the VR digitizer system. (A) Complete Vive hardware setup, with two controllers each communicating with the computer through the HMD, two trackers connected via independent wireless adapters, and all tracked devices receiving signals from two Lighthouses. (B) Reduced Vive hardware setup, with a single tracker and controller communicating through wireless adapters without an HMD. (C) Connections between software components.

The HTC Vive system includes an HMD, two tracked controllers, and two beacon devices called Lighthouses. These Lighthouses emit alternating horizontal and vertical sweeps of infrared light using a laser diode, in addition to interleaved synchronization flashes from an array of infrared LEDs. Multiple photodiodes on each tracked device (e.g. a controller) measure the relative perceived times of these infrared sweeps, allowing for 6DOF pose estimation. While some tracking can be achieved with just one Lighthouse, accuracy is improved if each tracked device can receive reference signals from more than one Lighthouse at the time of measurement. This can be ensured by proper room setup and tracker positioning. Each tracked device also has a 6DOF inertial measurement unit (IMU) to sense acceleration and rotational velocity, allowing for higher-temporal resolution relative pose tracking. Sensor fusion algorithms integrated into the Vive system merge the optical Lighthouse and IMU data together to calculate pose estimates that are passed on to the client software. In addition to the controllers, other tracked devices called “Vive Trackers” are available which include essentially the same tracking hardware without controls in a more compact form factor. The HMD is typically tethered to a computer, and up to two tracked devices can be paired to communicate wirelessly through the HMD, with additional devices using a wireless adapter that pairs the device to the computer (Fig 1A). Although the Vive system is primarily designed to be used with the HMD, individual controllers and trackers can be used without an HMD by pairing solely through wireless adapters (Fig 1B).

The primary goal of this work was to develop an open-source software package to facilitate digitization of EEG electrodes with cost-effective consumer VR hardware. We also aimed to characterize the typical digitization performance of such a system, and to compare it to other existing digitization systems.

## Methods

### VRDigitizer software

The primary functions for the VR digitizer software were implemented in MATLAB, with a small Python component mainly serving to relay raw device state data. The custom software and system we implemented are referred to as “VRDigitizer” here, with source code available at github.com/chriscline/VRDigitizer.

The Python component leverages PyOpenVR [17] and the OpenVR API [18] to obtain button state and tracking data for each device, including a rigid transformation matrix encoding position and orientation, and transmit these via TCP to MATLAB. With the Python component constantly streaming live tracking data to MATLAB, pressing a button on the controller triggers a sample. To mitigate the effects of high-frequency jitter in the raw Vive tracking data, VRDigitizer averages the tracking data over a window of time (0.5 sec). To deal with post-movement error persisting for up to several seconds after the end of controller movement, measurements are required to be stable (defined as being within an empirical threshold of 1 mm) over a window of time (1.0 sec). With each triggered sample, audio and haptic cues indicate to the user whether a measurement was deemed valid.

Electrode positions are typically defined relative to anatomical landmarks on the head, such as the nasion and preauricular points. These landmarks facilitate coregistration of electrode positions with MRI data and anatomical head models [19,20]. VRDigitizer allows specification of arbitrary fiducial points. Fiducials can be measured repeatedly to improve accuracy by averaging and to verify consistency across repeated measurements.

EEG montages can vary depending on the EEG cap vendor or other custom experiment requirements. VRDigitizer allows importing of many standard montage file formats, including optional specification of template electrode positions for visualization of “expected” electrode locations. During digitization, VRDigitizer provides optional audio cues through text-to-speech to indicate the next measurement to be made, and most functions can be carried out by pressing buttons on the controller; these features facilitate more efficient interaction with the GUI, allowing the operator to stay near the subject and reducing overall time for digitization.

The VRDigitizer GUI provides visualization of electrode positions relative to arbitrary surface meshes, such as scalp or cortical surfaces segmented from MRI data, with support for several common mesh formats (including .stl, .fsmesh, and .off). In the absence of a subject-specific head surface, an atlas head surface can be used by default.

During digitization, the controller is moved in 3D space to the location of a point to measure. For precise measurements, a single “endpoint” on the controller needs to be defined; this facilitates proper compensation for controller rotation around the endpoint. VRDigitizer allows specification of an arbitrary endpoint relative to the controller; to define this endpoint, a simple calibration routine is used. The user enters a calibration mode in the software and records several measurements across a range of controller rotations, keeping the intended endpoint fixed in space.

The calibration measurements are processed as follows. The $i^{th}$ sample from the PyOpenVR is structured as a 4×4 rigid spatial transform $T_{i}$ that converts from controller-relative space to a global space. Defining a point ${\overset{⃑}{x}}_{0}=[0001]$ as the origin of the controller, $T_{i}{\overset{⃑}{x}}_{0}$ gives the location of the center of the controller in global space. The goal of calibration is to find ${\overset{⃑}{x}}_{c}$ in the device-relative space that defines the offset of the intended endpoint relative to the ${\overset{⃑}{x}}_{0}$. The global position ${\overset{⃑}{x}}_{g}$ of the endpoint for the $i^{th}$ measurement can then be calculated from $T_{i}\left({\overset{⃑}{x}}_{0}+{\overset{⃑}{x}}_{c}\right)$. Given a set of N spatial transforms $\left\{T_{i}\right\}$, each from a measurement with a different controller orientation around the shared fixed endpoint, ${\overset{⃑}{x}}_{c}$ can be obtained through numerical optimization. Specifically, the optimization problem can be expressed as:

$${\text{argmin}}_{{\vec{x}}_{c}}\sum_{i=1}^{N}{\left|\underset{{\vec{x}}_{gi}}{\underbrace{T_{i}\left({\vec{x}}_{0}+{\vec{x}}_{c}\right)}}-\frac{1}{N}\sum_{j=1}^{N}\underset{{\vec{x}}_{gj}}{\underbrace{T_{j}\left({\vec{x}}_{0}+{\vec{x}}_{c}\right)}}\right|}_{2}$$ (1)

The solution to this expression is the ${\overset{⃑}{x}}_{c}$ that minimizes the spread of $\left\{{\overset{⃑}{x}}_{g}\right\}$ derived from the calibration measurements. Metrics of calibration quality can be obtained from terms of this cost function, such as the maximum deviation from the mean:

$$\max_{i\in N}{\left|{\vec{x}}_{gi}-\frac{1}{N}\sum_{j=1}^{N}{\vec{x}}_{gj}\right|}_{2}$$ (2)

**Fig 2:**
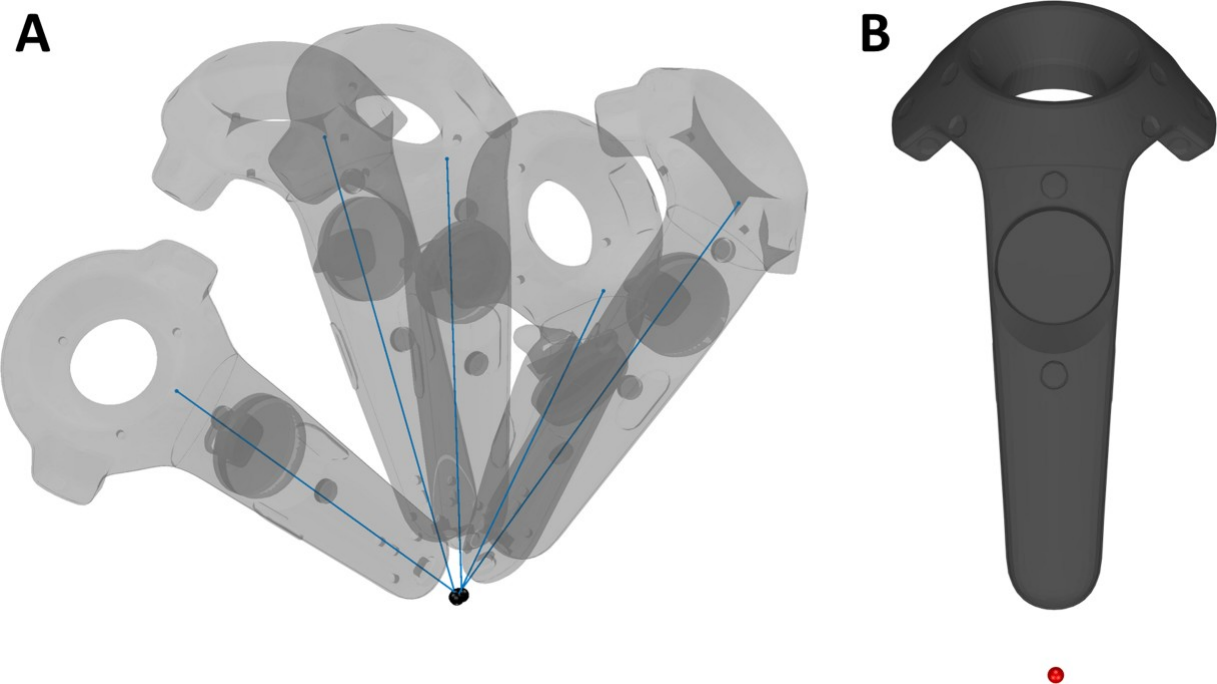
Example of endpoint calibration. (A) Visualization of calibration process, showing five samples measured with varied rotations about the selected endpoint. Black spheres indicate the estimated endpoint location for each sample, and blue lines connect the estimated endpoint to the controller origin. (B) Final endpoint location relative to controller. The endpoint (red sphere) is offset from controller since it was defined as a point at the end of a micro USB cable stub not included in the controller 3D model.

VRDigitizer solves Eq (1) using constrained nonlinear optimization as implemented by ‘fmincon’ in MATLAB. The results of calibration are shown by VRDigitizer with a depiction of the estimated endpoint position relative to the controller and a visualization of individual calibration measurements; an example of this is shown in Fig 2.

During electrode digitization, the subject’s head may move over time. If there is no compensation for this change in position, error is introduced between the measurements before and after a given movement. One solution to this is to attempt to fix the subject’s head during digitization measurements, thus minimizing movement. However, this may not be feasible for many experimental setups based on subject comfort and other factors. The solution more commonly used by EEG digitization systems is to simultaneously track head position in addition to the measurement stylus. VRDigitizer can perform this head tracking using standalone tracked devices fixed to the subject’s head. While the HMD is designed for head tracking and is bundled with the core Vive hardware, its straps are likely to block many electrodes and the faceplate itself blocks the nasion, an anatomical landmark typically measured during digitization. VRDigitizer can instead use standalone Vive Trackers to measure head movement. When enabled, VRDigitizer measures the controller endpoint relative to one or more trackers, allowing compensation for head movement in real-time.

Many EEG digitization systems use a single additional tracking device for head movement compensation. However, if this head tracker moves relative to the head during measurement, additional error can be introduced. With only a single tracker on the head, this problem may go undetected during measurement. VRDigitizer allows the use of multiple head trackers; if one tracker moves on the head, the mismatch in trackers can be detected and a warning can notify the user to realign the trackers or remeasure fiducials with the new tracker positions.

In addition to digitization of anatomical fiducials and electrodes, arbitrary points on the head surface can also be sampled. These points may be used to assist with MRI coregistration or atlas warping during post-processing [19,20].

**Fig 3:**
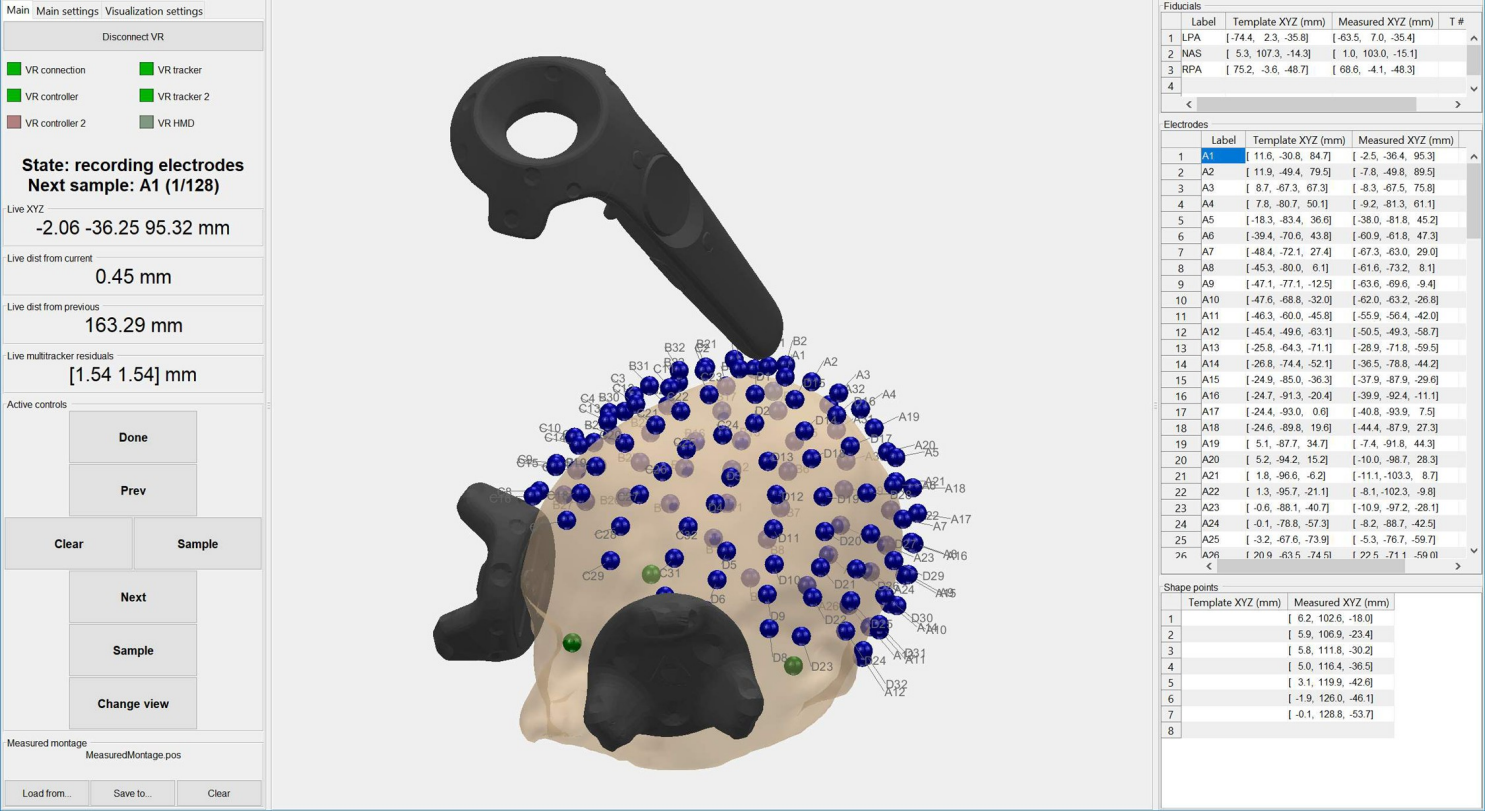
Screenshot of main VRDigitizer GUI window.

VRDigitizer can provide real-time visualization of various components, including the head surface, template montage, measured fiducials, measured electrodes, and endpoint, controller, and tracker positions. This is illustrated in Fig 3, which shows an example screenshot from the main VRDigitizer GUI window.

### Experimental evaluation

To characterize the expected performance of the VRDigitizer setup and compare it to other digitization methods, two sets of experiments were conducted. The first set involved a “phantom” styrofoam head, while the second involved 12 human subjects who provided written informed consent under a protocol approved by the University of Minnesota Institutional Review Board. In both series of experiments, BioSemi 128 channel caps (BioSemi B.V., Amsterdam, Netherlands) with a radial montage were used.

In addition to VRDigitizer, two other systems were utilized for digitization performance comparison. The first was a Brainsight neuronavigation system (Rogue Research, Montreal, Canada), which uses a Polaris Vicra stereo infrared camera (NDI, Waterloo, Canada) to track infrared reflective spheres fixed on rigid frames for monitoring the pose of a stylus relative to safety glasses on the subject’s head. The second system was a FASTRAK (Polhemus, Colchester, VT, USA) utilizing the open-source Brainstorm software [19]; this system uses radio-frequency (RF) electromagnetic tracking with a stationary RF transmitter and two 6DOF tracked receivers, one in a stylus and another on the subject’s head.

Two different variations of VRDigitizer setups were used: digitization with no trackers (i.e. no compensation for head movement), and with two head trackers. In the no tracker condition, the phantom head was secured to a rigid support, and human subjects used a chinrest and forehead support attached to a table to minimize head movement; no HMD was connected, instead a single wireless adapter was used to connect to the controller. In the two-tracker condition, Vive trackers were secured with a Velcro strap to either side of the forehead (as illustrated in Fig 3); the controller and both trackers were connected via the HMD and a single wireless adapter.

Vive Lighthouses were attached to the wall in diagonally opposite corners of the room (4.25 m apart, 2.6 m above the floor), and were synchronized by line-of-sight infrared communication. A stub of a microUSB cable 27 mm in length with a tip 3 mm in diameter was inserted into the base of the controller to provide a precise digitization endpoint; this location of the endpoint at the base of the controller (as shown in Fig 2) also helped to ensure the photodiode sensors at the top of the controller maintained a view of both Lighthouses during digitization.

The VRDigitizer calibration interface was used to calibrate the endpoint position relative to the center of the controller. Approximately 10 measurements were sampled with the controller rotated into different orientations around the selected endpoint, which was held at a fixed point in space throughout calibration.

With each subject or phantom iteration, 5 whole-head digitizations were performed, consisting of VRDigitizer without trackers (“NoTrackers”), VRdigitizer with two trackers (“TwoTrackers”), Polhemus with Brainstorm (“Brainstorm”), and two Brainsight repetitions. The VRDigitizer NoTrackers condition included head restraint, while the other conditions did not. Subjects donned the cap once and kept the cap in place throughout the 5 digitizations, for a total duration of about 50–60 minutes.

For each digitization dataset collected with each system, several steps were performed. Head trackers (as applicable) were fixed to the head. Anatomical fiducials were digitized first; with the same nasion and left and right preauricular points measured with each system. Next, electrodes were digitized, always proceeding in order from BioSemi electrodes A1,A2, … to D32; each electrode on the BioSemi cap included a printed label, which was used to define a point < 2 mm in diameter as a consistent target for digitization. Finally, each fiducial was re-measured twice as a head shape point.

### Analysis

To estimate localization accuracy, digitization errors were calculated using one Brainsight dataset in each subject (or phantom repetition) as the reference or “ground truth”. The first Brainsight dataset in each subject (or phantom repetition) was used as reference by default. In rare cases of operator error of digitizing an incorrect electrode (e.g. pointing to “A2” when prompted to point to “A1”), identified by visual inspection, individual electrode measurements were rejected and not included for alignment or accuracy calculations. This resulted in rejection of at most 2 electrodes per dataset, with a total of 7 electrode measurements rejected across all datasets (<0.06% of all measurements). In cases where the first Brainsight dataset for a subject had more rejected electrodes than the second Brainsight dataset, the latter was used as reference. See S1 Fig for a supplementary evaluation using other datasets as references for alignment.

Each dataset was aligned to the reference by estimating and applying a single rigid spatial transformation that best aligned paired sets of points across the datasets [20]. These alignment points were either the anatomical fiducials only (i.e. aligning by fiducials) or the measured electrode locations (i.e. aligning by electrodes). The former case is representative of common use of EEG digitization data in practice, in which as a first stage of co-registration with MRI the digitized points are aligned by the corresponding anatomical fiducial locations in the MRI [19]. However, in this case of aligning by fiducials, inconsistency in a single fiducial location can propagate and appear as error in localization across all electrodes on the head. Therefore, the second case of aligning by electrodes was used to provide an estimate of electrode localization accuracy independent of anatomical fiducial measurements.

After alignment, localization error was quantified as the Euclidean distance between each electrode and its corresponding reference location. Statistical comparisons were calculated using Welch’s two-tailed t-tests without correction for multiple comparisons.

## Results

**Fig 4:**
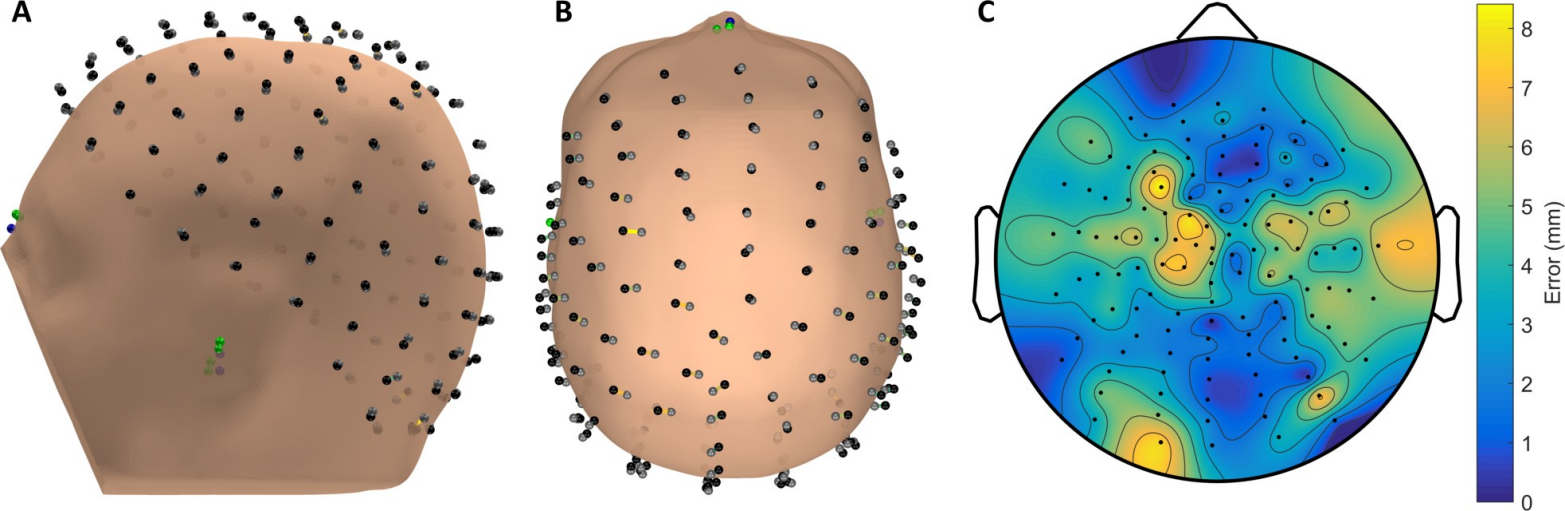
VRDigitizer localization results for a single subject (S06). In (A) and (B), the green and gray circles indicate fiducials and electrodes measured for the VRDigitizer TwoTrackers condition, while the blue and black circles indicate fiducials and electrodes measured for the Brainsight reference condition; the colors of the lines connecting corresponding electrodes across the two datasets are indicative of the magnitude of localization error. Interpolated localization error is plotted with the same color scale on a projected 2D scalp topography in (C). Here, the two datasets were aligned by electrodes, and the subject’s scalp surface was aligned by the reference dataset anatomical fiducials. Electrodes appear offset from the scalp surface due to the selected digitization point for each electrode being on the top surface of the 3-mm thick electrode mount.

Example digitization results using VRDigitizer with two trackers for a single subject are shown in Fig 4. Aligned to the reference Brainsight digitization dataset by electrodes, the RMS localization error here was 3.95 mm, and the maximum error was 8.41 mm. For comparison, most neighboring electrodes in the BioSemi 128 radial montage used here had interelectrode distances of between 18–25 mm for this subject.

**Fig 5:**
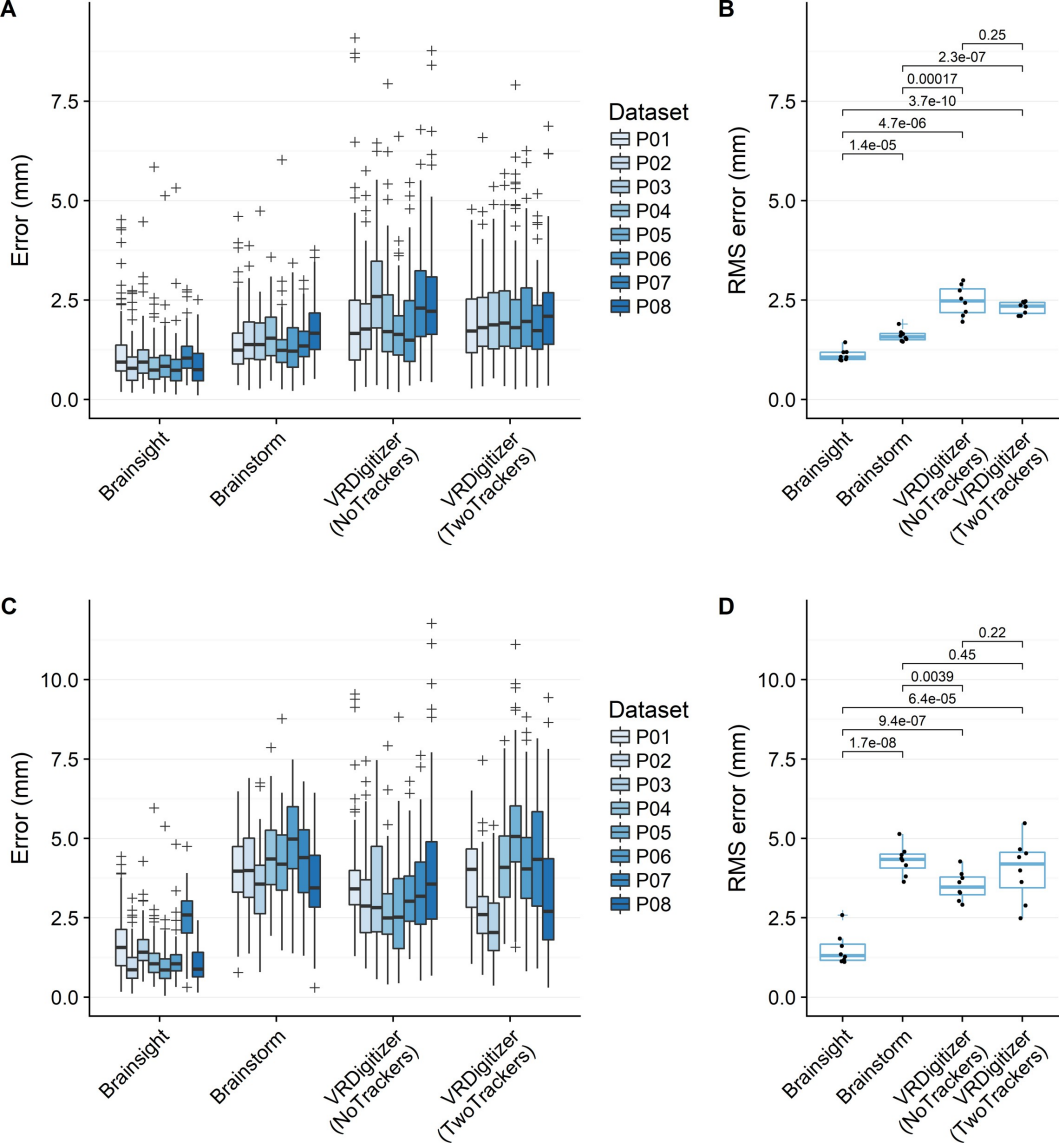
Localization errors for phantom measurements. Aligned by electrodes (A,B) or by fiducials (C,D). Individual results are shown in (A) and (C), with each box-and-whisker corresponding to a single dataset and each point corresponding to localization error for a single electrode. Grouped results are shown in (B) and (D), with each point corresponding to a scalar RMSE error aggregated from a single dataset. Labeled values in (B) and (D) indicate *p* values from uncorrected two-tailed Welch’s t-tests.

Fig 5 shows localization errors for multiple digitizations of the phantom head. Aggregating errors within each dataset by the root mean square error (RMSE), mean ± standard deviation RMSE values when aligning by electrodes were 1.12 ± 0.15 mm for Brainsight, 1.60 ± 0.15 mm for Brainstorm, and 2.30 ± 0.16 mm for VRDigitizer with two trackers. Brainsight demonstrated significantly lower errors than the other systems. Brainstorm demonstrated significantly lower errors than both VRDigitizer conditions when aligning by electrodes but not when aligning by fiducials.

**Fig 6:**
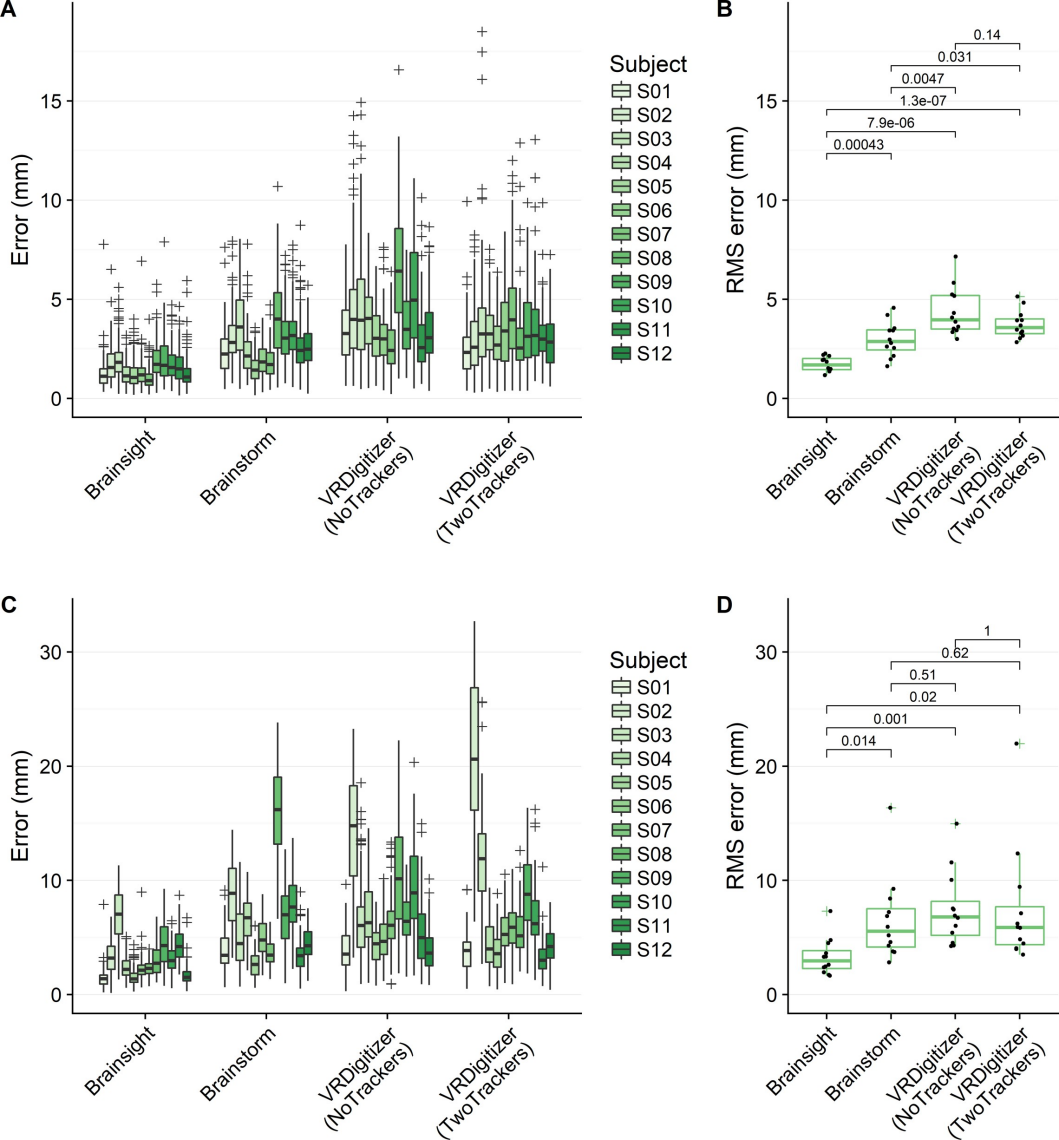
Localization errors for subject measurements. Aligned by electrodes (A,B) or by fiducials (C,D). Individual results are shown in (A) and (C), with each box-and-whisker corresponding to a single dataset and each point corresponding to localization error for a single electrode. Grouped results are shown in (B) and (D), with each point corresponding to a scalar RMSE error aggregated from a single dataset. Labeled values in (B) and (D) indicate *p* values from uncorrected two-tailed Welch’s t-tests.

Similar trends were observed for localization errors with human subjects, with results shown in Fig 6. Aligning by electrodes, mean ± standard deviation RMSE values were 1.73 ± 0.37 mm for Brainsight, 2.98 ± 0.89 mm for Brainstorm, and 3.74 ± 0.71 mm for VRDigitizer with two trackers.

## Discussion

We have developed a new digitizer system, allowing digitization of EEG electrode positions in 3D space using widely-available consumer virtual reality hardware. The estimated accuracy of VRDigitizer is comparable to existing digitization systems, with a typical RMS localization error of less than 5 mm.

Critically, the digitization system described here uses commercial virtual reality hardware available for approximately $500-$700 at current prices; the software is free and open-source. Other systems for digitization of EEG electrodes can cost 10–20 times this amount, limiting the number of EEG research projects able to include digitization of electrode positions.

Despite cost benefits, the described VRDigitizer system did not match the performance of the Brainsight/Polaris and Brainstorm/Polhemus systems tested here. Each of the three systems use different tracking methods. Brainsight uses an infrared stereo camera with fixed geometry passive reflective trackers; this can be very precise but prone to problems with optical occlusion between the single mounted camera and the reflective trackers. Brainstorm uses an RF transmitter and receivers which do not require line of sight; however, this system can be subject to geometric distortion in the presence of nearby metal objects (see supplementary S2 Fig). VRDigitizer with the Vive estimates tracked device pose by measuring the relative timing of signals emitted from the shared Lighthouse beacons, combined with IMU data; the Vive also can have issues with optical occlusion. Additionally, the rigidity and precision of the stylus tip and the related endpoint calibration can contribute to overall system accuracy.

For the results presented here, the electrode positions measured by the Brainsight system were used as the “ground truth” or reference for estimating localization accuracies. The relatively low RMS errors observed between repeated Brainsight measurements, and comparisons with using other systems as reference (S1 Fig) support the assumption that this system provides a suitable reference with which to compare the various digitization systems. However, it is possible that some consistent bias or geometric distortion was present in these repeated measurements. Effects of such consistent error, if any, were minimized during measurements by moving the head relative to the Brainsight/Polaris camera, repositioning the head tracking glasses between repeated sets of measurements, and using slightly different stylus orientations across repeated measurements. Nevertheless, more rigorous measurements of digitization accuracy could be obtained in the future by using a phantom with precise, rigidly defined points (e.g. as used in [12]).

Each system tested here still requires the operator to “point” a stylus at each electrode during digitization; the amount of time required for this process can discourage the use of digitization in some cases. One possible method for reducing time needed for digitization with stylus-based systems is to only digitize a representative subset of electrodes [15]. While not yet implemented here, the open-source VRDigitizer software can be freely modified, and such improvements can therefore be integrated into the software in the future.

Other recently introduced systems using photogrammetry [12,21] or 3D scanners [14] hold promise for faster and more automatic electrode digitization with comparable accuracy. While promising, most of these approaches also require hardware that is currently more expensive than the VRDigitizer system. One recently described approach with similar hardware cost uses a consumer-grade camera to take images of a subject wearing a cap from multiple angles, applies photogrammetry software to reconstruct a 3D mesh of the head, then extracts electrode positions from the mesh [21]. This approach has several possible advantages compared to VRDigitizer, including reduced measurement error and faster data acquisition. However, in the approach described in [21], expensive proprietary software is used for mesh reconstruction, images of a subject’s face are required, reconstruction quality is sensitive to lighting conditions, and no feedback on quality of digitization data is provided during acquisition.

Overall, the availability of cost-effective hardware and open source software for digitization of EEG electrode positions should benefit the EEG community, facilitating analysis of EEG signals in source space using inverse source imaging methods, with more relevant subject-specific head models and electrode positions. Such approaches are necessary to move beyond single channel analyses and scalp topographies for more robust individual and group-level EEG source imaging.

## Supporting information

S1 Fig Aggregated localization errors as a function of selected reference dataset. Results of aligning to each dataset as reference in turn within each subject (or phantom repetition), with remaining datasets aligned to the specified reference by electrodes (A,B) or fiducials (C,D), aggregated over phantom repetitions (A,C) or subjects (B,D). These results supplement those presented in the main article, in which a single Brainsight dataset was used as reference within each subject (or phantom repetition).
(TIF)

S2 Fig Example of distortion in Brainstorm/polhemus measurements caused by nearby metal. A 40 mm x 40 mm x 300 mm piece of extruded aluminum was placed oriented vertically near the phantom head to demonstrate the effects of the presence of metal on Polhemus digitizer measurements. The RF transmitter was approximately 22 cm from the center of the head along the horizontal plane, and approximately 10 cm below the lowest electrode. (A) shows the results of digitizing with the aluminum piece placed between the RF transmitter and the of the head, about 7 cm from the center of the head. (B) and (C) show the results of digitizing after moving the aluminum piece 10 cm and 20 cm to the right (perpendicular to the line between the RF transmitter and the center of the head), respectively. (D) shows the results of digitizing without any metal nearby with the Polhemus system. (E) shows example results of digitizing with the VRDigitizer two tracker setup. In the upper and middle plots, the green and gray circles indicate fiducials and electrodes measured by the Polhemus digitizer (A-D) or VRDigitizer (E), while the blue and black circles indicate fiducials and electrodes measured for the Brainsight reference condition; the colors of the lines connecting corresponding electrodes across the two datasets are indicative of the magnitude of localization error. Interpolated localization error is plotted with the same color scale on a projected 2D scalp topography in the lower plots.
(TIF)
