# Supplementary figures and images for: EEG electrode digitization with commercial virtual reality hardware
Source: PLoS One. 2018 Nov 21;13(11):e0207516. doi: 10.1371/journal.pone.0207516 (PMC6248988; doi:10.1371/journal.pone.0207516)

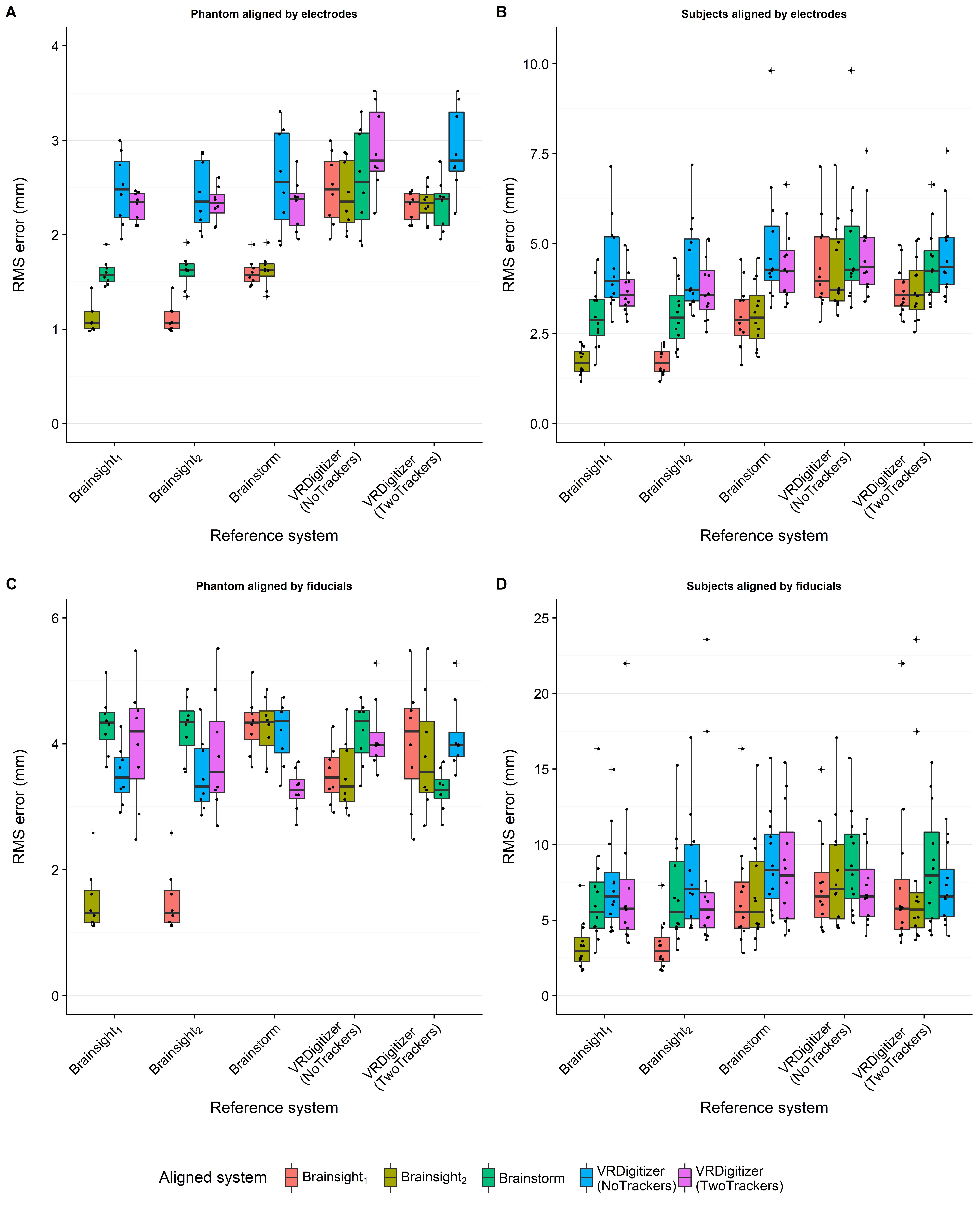

Supplement: S1 Fig — Results of aligning to each dataset as reference in turn within each subject (or phantom repetition), with remaining datasets aligned to the specified reference by electrodes (A,B) or fiducials (C,D), aggregated over phantom repetitions (A,C) or subjects (B,D). These results supplement those presented in the main article, in which a single Brainsight dataset was used as reference within each subject (or phantom repetition). (TIF) [file pone.0207516.s001.tif]

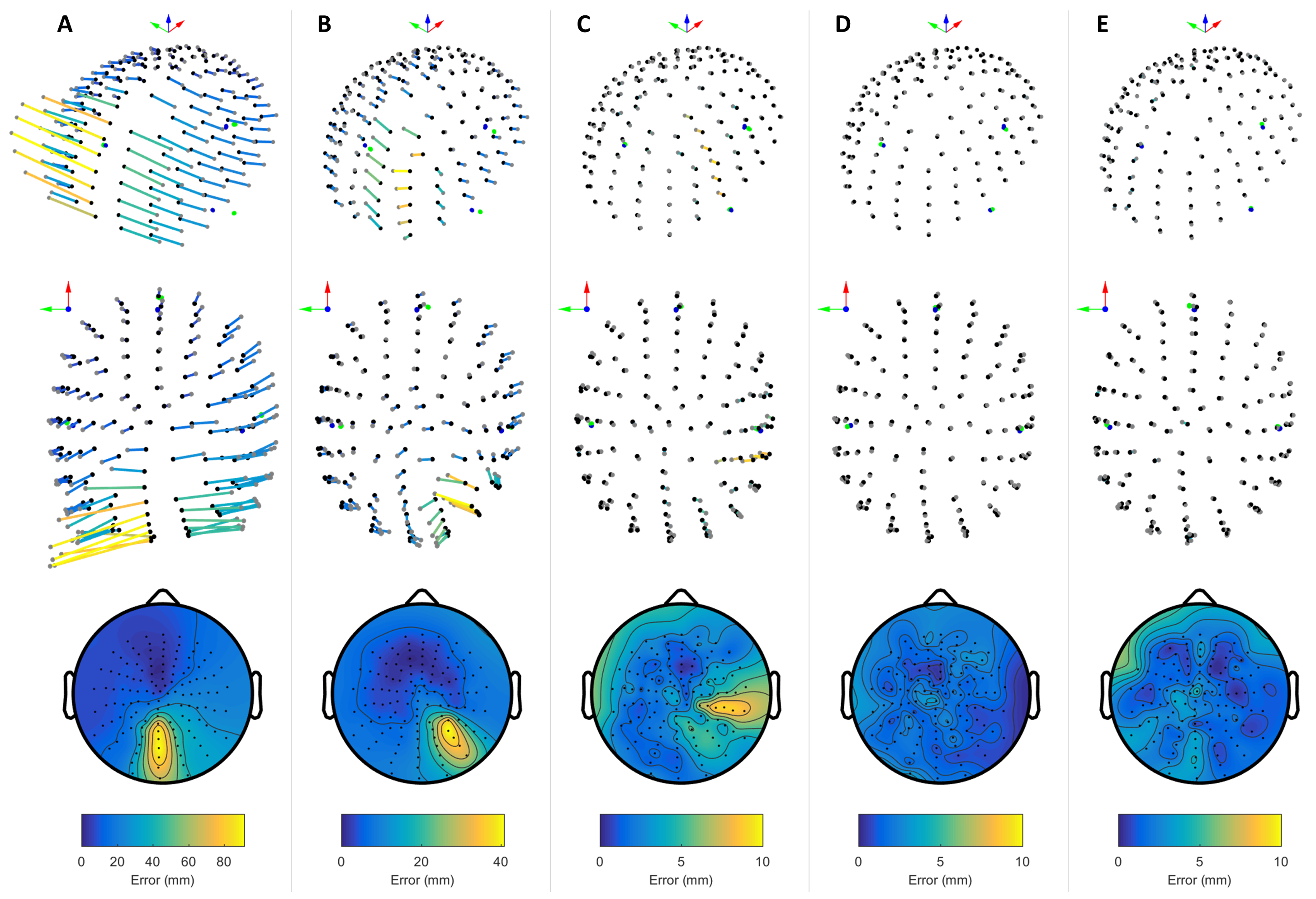

Supplement: S2 Fig — A 40 mm x 40 mm x 300 mm piece of extruded aluminum was placed oriented vertically near the phantom head to demonstrate the effects of the presence of metal on Polhemus digitizer measurements. The RF transmitter was approximately 22 cm from the center of the head along the horizontal plane, and approximately 10 cm below the lowest electrode. (A) shows the results of digitizing with the aluminum piece placed between the RF transmitter and the of the head, about 7 cm from the center of the head. (B) and (C) show the results of digitizing after moving the aluminum piece 10 cm and 20 cm to the right (perpendicular to the line between the RF transmitter and the center of the head), respectively. (D) shows the results of digitizing without any metal nearby with the Polhemus system. (E) shows example results of digitizing with the VRDigitizer two tracker setup. In the upper and middle plots, the green and gray circles indicate fiducials and electrodes measured by the Polhemus digitizer (A-D) or VRDigitizer (E), while the blue and black circles indicate fiducials and electrodes measured for the Brainsight reference condition; the colors of the lines connecting corresponding electrodes across the two datasets are indicative of the magnitude of localization error. Interpolated localization error is plotted with the same color scale on a projected 2D scalp topography in the lower plots. (TIF) [file pone.0207516.s002.tif]
